# AZD1390, an ataxia telangiectasia mutated inhibitor, attenuates microglia‐mediated neuroinflammation and ischemic brain injury

**DOI:** 10.1111/cns.14696

**Published:** 2024-04-26

**Authors:** Zhen Lan, Long‐jie Qu, Ying Liang, Li‐qiu Chen, Shuai Xu, Jian‐wei Ge, Zhi‐wei Xue, Xin‐yu Bao, Sheng‐nan Xia, Hai‐yan Yang, Jing Huang, Yun Xu, Xiao‐lei Zhu

**Affiliations:** ^1^ Department of Neurology Nanjing Drum Tower Hospital, Clinical College of Nanjing Medical University Nanjing Jiangsu China; ^2^ Department of Neurology Nanjing Drum Tower Hospital Clinical College of Nanjing University of Chinese Medicine Nanjing Jiangsu China; ^3^ Department of Neurology Nanjing Drum Tower Hospital, The Affiliated Hospital of Nanjing University Medical School Nanjing Jiangsu China; ^4^ State Key Laboratory of Pharmaceutical Biotechnology and Institute of Translational Medicine for Brain Critical Diseases Nanjing University Nanjing Jiangsu China; ^5^ Jiangsu Key Laboratory for Molecular Medicine Medical School of Nanjing University Nanjing Jiangsu China; ^6^ Nanjing Neuropsychiatry Clinic Medical Center Nanjing Jiangsu China

**Keywords:** ATM inhibitor, AZD1390, ischemic stroke, microglia, neuroinflammation, NF‐κB pathway

## Abstract

**Aims:**

Excessive neuroinflammation mediated mainly by microglia plays a crucial role in ischemic stroke. AZD1390, an ataxia telangiectasia mutated (ATM) specific inhibitor, has been shown to promote radio‐sensitization and survival in central nervous system malignancies, while the role of AZD1390 in ischemic stroke remains unknown.

**Methods:**

Real‐time PCR, western blot, immunofluorescence staining, flow cytometry and enzyme‐linked immunosorbent assays were used to assess the activation of microglia and the release of inflammatory cytokines. Behavioral tests were performed to measure neurological deficits. 2,3,5‐Triphenyltetrazolium chloride staining was conducted to assess the infarct volume. The activation of NF‐κB signaling pathway was explored through immunofluorescence staining, western blot, co‐immunoprecipitation and proximity ligation assay.

**Results:**

The level of pro‐inflammation cytokines and activation of NF‐κB signaling pathway was suppressed by AZD1390 in vitro and in vivo. The behavior deficits and infarct size were partially restored with AZD1390 treatment in experimental stroke. AZD1390 restrict ubiquitylation and sumoylation of the essential regulatory subunit of NF‐κB (NEMO) in an ATM‐dependent and ATM‐independent way respectively, which reduced the activation of the NF‐κB pathway.

**Conclusion:**

AZD1390 suppressed NF‐κB signaling pathway to alleviate ischemic brain injury in experimental stroke, and attenuated microglia activation and neuroinflammation, which indicated that AZD1390 might be an attractive agent for the treatment of ischemic stroke.

## Graphical abstract

AZD1390 suppressed NF‐κB signaling pathway through restricting ubiquitylation and sumoylation of NEMO, which alleviated ischemic brain injury in experimental stroke and attenuated the activation of microglia and neuroinflammation.

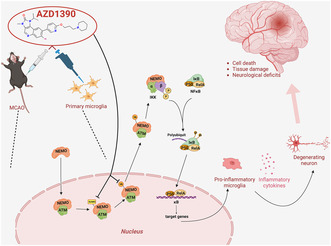

## 1 INTRODUCTION

Stroke is the second‐leading cause of death in the world and ischemic stroke accounts for the most part.^1^ The high mortality and the rate of disability threaten human health and quality of life. Currently, thrombolysis and endovascular thrombectomy have become the efficacious treatments of ischemic stroke, but the narrow therapeutic window and attendant risks hinder the clinical application. Therefore, more feasible and efficient therapy is pressing needed.

Cumulative evidence suggests that ischemic stroke triggers a profound neuro‐inflammatory response, thereinto, the microglia play a pivotal role in the pathogenesis of ischemic stroke.^2^ Due to the sudden blockage of cerebral vessel, multiple factors are released to stimulate the activation and migration of nearby microglia to protect the brain from ischemic stroke. However, activated microglia also sparks the inflammation pathways to exacerbate brain injury. Reactive oxygen species (ROS) responsive polymer‐drug conjugate nanoparticles are developed to suppress microglia polarization to reduce post‐stroke inflammation.^3^ Peroxiredoxin‐1, predominantly expressed in stroke‐associated microglia, increases the transcription levels of stroke‐protective molecules and exerts neuroprotective functions.^4^ Our previous data have shown that imperatorin inhibits the activation of microglia and protects against ischemic stroke.^5^ Collectively, precise modulation of microglia functions emerges as a promising therapeutic strategy for ischemic stroke.

AZD1390, a highly selective ataxia telangiectasia mutated (ATM) inhibitor, is orally bio‐available and blood–brain barrier penetrant, and is considered as a candidate drug for brain malignancies.^6^ The Phase 1 study of AZD1390 with radiation treatment in glioblastoma and brain metastases is undergoing (NCT03423628). While sensing the stimulators, the essential regulatory subunit of NF‐κB (NEMO) translates into nuclear via small ubiquitin‐like modifier 1 (SUMO‐1) attachment, and ATM is activated to mediate the subsequent ubiquitylation of NEMO. Sequentially, NEMO translocates to cytoplasm to phosphorylate IKK complex and ultimately activates the NF‐κB pathway.^7^ However, little is known about the effect of AZD1390 in ischemic stroke. In this study, we aimed to explore the effects and potential mechanisms of AZD1390 on neuroinflammation and microglial activation in ischemic stroke, which might reveal a potential compound for the treatment of stroke.

## 2 MATERIALS AND METHODS

### 2.1 Materials

AZD1390 (CAS:2089288‐03‐7, Purity: 99.97%) was purchased from MedChemExpress (Shanghai, China) and dissolved in dimethyl sulfoxide (DMSO, BS087, Biosharp) for the in vivo and in vitro experiments. The concentration of DMSO was controlled to below 1‰ to avoid its toxic side effects. Lipopolysaccharide (LPS from *Escherichia coli* 055: B5, tlrl‐pb5lps, InvivoGen) was obtained from InvivoGen (San Diego, CA, USA).

### 2.2 Cell culture

Primary microglia were extracted from newborn C57/BL6J mice and cultured in Dulbecco's modified Eagle's medium (11995065, Thermo Fisher) with 10% fetal bovine serum (FBS, 10099‐141, Thermo Fisher Scientific) and 1% antibiotics at 37°C in a humid atmosphere containing 5% CO_2_ for 10–12 days. The purify of primary microglia was determined by immunostaining with Iba1 antibody (ab5076, Abcam). Microglia were pretreated with AZD1390 (0.5, 2 or 10 μM) for 2 h and then stimulated with LPS (100 ng/mL) for 24 h.

### 2.3 Animals and middle cerebral artery occlusion (MCAO)

Male 8‐week‐old C57BL/6 mice were provided by Nanjing University Animal Model Center (Nanjing, Jiangsu, China), and MCAO model was performed as previously described.^5^ All animal experiments were granted permission by the Animal Care and Use Committee at Nanjing Drum Tower Hospital. Mice were settled in cages with a light cycle of 12‐h light/12‐h dark. Mice were randomly divided into three groups: sham‐operate (SHAM) group, 2% DMSO‐treated MCAO (DMSO) group, and AZD1390 (2, 5 or 10 mg/kg)‐treated MCAO group. Mice weighed 20–25 g were anesthetized with Avertin (T48402, Sigma‐Aldrich). After the right internal carotid artery (ICA) and the middle cerebral artery (MCA) were exposed well, a piece of 6/0 surgical suture with a heat‐rounded tip (Doccol Corporation, MA, USA) was inserted into the MCA until its blood flow dropped to 30% of baseline. The blood flow was monitored through Laser Doppler flowmetry (Perimed Corporation, Stockholm, Sweden). One hour later, the filament was withdrawn to re‐open blocked blood vessels. The sham‐operate group underwent the same surgery except the suture inserting. All mice were maintained at 37 ± 0.5°C on a heating pad after operation. The mice were intraperitoneal injected with AZD1390 (2, 5 or 10 mg/kg) or DMSO (2%) at 30 min, 24 h, and 48 h post‐reperfusion.

### 2.4 Neurobehavioral tests

Before the MCAO modeling, mice were trained for 3 days, and baseline of modified neurological severity scores (mNSS) test, the rotarod experiment, the forelimb grip strength experiment and footfault test was recorded. All these tests were also performed at 24 and 72 h after reperfusion.

The mNSS test reflects the neurological function including motor, sensory, balance and reflexes aspects. The higher score indicates severer neurological deficits.

The rotarod experiment recorded the level of sensorimotor coordination and balance. Mice were test in the accelerating rotarod (from 10 to 40 rpm) for 5 min and recorded the holding time at the maximum speed. Specifically, mice should rest for 10 min to continue the subsequent stages.

Forelimb grib strength was accessed by a grip strength meter (GS3, Biosed, France). Briefly, the mice were held by the tail and their forepaw could grasp the strength rodmeter, avoiding the interference of body weight. The maximum value was recorded and analyzed.

### 2.5 Infarct volume measurement

Brains were taken out at 72 h after operation and cut into seven slices (1 mm thick) and soaked in 2,3,5‐triphenyltetrazolium chloride (TTC, T8877‐100G, Sigma‐Aldrich) until the brain slices were colored. Then the slices were analyzed with ImageJ (ImageJ 1.5, NIH). The percentage of infarct volume was calculated as (contralateral hemisphere volume − ipsilateral normal volume)/(2 × contralateral volume) × 100%.

### 2.6 Enzyme‐linked immunosorbent assay

The supernatant containing cytokines was collected from treated primary microglia and analyzed through the IL‐1β, IL‐6, and TNF‐α ELISA kits according to the manufacturer's instructions (CEK1731/CEK1737/CEK1744, Bioworld Biotechnology).

### 2.7 Immunofluorescence

Mice brains were gained to fix in 4% paraformaldehyde and dehydration with graded sucrose. After that, the brain was cut into 20 μm through a cryostat microtome (Leica, Wetzlar, Germany). Brain slices or primary microglia were moistened in 3 times with 1 × PBS and permeabilized with 0.25% Triton X‐100 for 20 min, and then blocked with 2% BSA (4240GR250, BioFroxx) for 2 h and incubated with primary antibody: anti‐Iba1 (ab5076, Abcam), anti‐P65 (8242, Cell Signaling Technology), or anti‐ATM (ab32420, Abcam) overnight at 4°C. Subsequently, microglia or slices were immersed in secondary antibody: Alexa 488 goat anti‐Iba1, Alexa 594 rabbit anti‐P65 or Alexa 647 rabbit anti‐ATM (A32814/A32754/ A32795, Invitrogen) for 1–2 h at 37°C. The nuclear was stained with DAPI (BD5010, Bioworld Biotechnology) for 10–15 min. Images were obtained by Olympus BX51 (Japan) fluorescence microscope and analyzed with ImageJ (ImageJ 1.5, NIH).

### 2.8 Western blot

Protein from microglia or ischemic penumbra was centrifuged at 13,000 × g at 4°C for 30 min and quantified through a BCA kit (P0011, Beyotime Biotech). Equal amounts of protein were loaded onto 10% SDS‐PAGE gel (PG212, Epizyme), separated by electrophoresis and transferred to Polyvinylidene fluoride membranes (PVDF, 1620177, Bio‐Rad). The membrane was blocked by 5% non‐fat milk for 2 h and incubated with primary antibodies against IL‐1β (sc52012, Santa Cruz), IL‐6 (sc‐28343, Santa Cruz), TNF‐α (sc‐52746, Santa Cruz), iNOS (BS40374, Bioworld Biotechnology), p‐NF‐κB/p‐P65 (3033, Cell Signaling Technology), NF‐κB/P65 (8242, Cell Signaling Technology), IKKα/β (sc‐8032, Santa Cruz), p‐IKKα/β (2697, Cell Signaling Technology), IκBα (BS3601, Bioworld Biotechnology), p‐IκBα (2859, Cell Signaling Technology), NEMO (ab178872, abcam), anti‐Ubiquitin (ab134953, abcam), anti‐SUMO (ab32058, abcam), COX‐2 (BS1076, Bioworld Biotechnology) or β‐actin (BS40736, Bioworld Biotechnology) overnight at 4°C. On the following day, the membrane was immersed in the corresponding secondary antibodies (BS22357/BS22356, Bioworld Biotechnology) on the shaker for 1–2 h. After moistened with the enhanced chemiluminescence (ECL, 34580, Thermo Fisher Scientific), the protein band was visualized through Gel‐Pro system (Tanon Technologies) and analyzed using ImageJ (ImageJ 1.5, NIH).

### 2.9 Quantitative real‐time PCR (qPCR)

Total RNA samples were extracted by TRIzol reagent (Invitrogen Life Technologies, Carlsbad, CA, USA) and reversed transcription was performed according to the protocol using PrimeScript RT Master Mix (Vazyme, Nanjing, China). Consequently, the qPCR was conducted with the SYBR Green kit (Applied Biosystems) and the Step One Plus PCR system (Applied Biosystems).

The primer sequences are shown below:

IL‐1β:
  Forward, CCATCCTCTGTGACTCATGGG;
  Reverse, TCAGCTCATATGGGTCCGAC;
IL‐6:
  Forward, GACAAAGCCAGAGTCCTTCAGAGAG;
  Reverse, CTAGGTTTGCCGAGTAGATCTC;
TNF‐α:
  Forward, CCACCACGCTCTTCTGTCTA;
  Reverse, GATCTGAGTGTGAGGGTCTGG;
iNOS:
  Forward, CACCACCCTCCTCGTTC;
  Reverse, TGCCTATCCGTCTCGTC;
COX‐2:
  Forward, TGCTGGTGGAAAAACCTCGT;
  Reverse, AAAACCCACTTCGCCTCCAA;
CD86:
  Forward, CCAGAACTTACGGAAGCACC;
  Reverse, CCAGAACACACACAACGGTC;
Arg‐1:
  Forward, AGCACTGAGGAAAGCTGGTC;
  Reverse, TACGTCTCGCAAGCCAATGT;
GAPDH:
  Forward, AGGTCGGTGTGAACGGATTTG;
  Reverse, TGTAGACCATGTAGTTGAGGTCA.

### 2.10 Flow cytometry

The penumbra tissue was obtained 3 days after MCAO and made into single cells suspension, and then incubated with CD45‐PECy7 (25‐0451‐82, eBioscience) and CD86‐BV421 (62‐0869‐42, eBioscience) for 30 min at 4°C. Cells were detected via an FACS Calibur flow cytometer (BD Biosciences) and further analyzed with FlowJo‐V10 software.

### 2.11 Co‐immunoprecipitation (IP)

Protein was extracted from ischemic penumbra with or without AZD1390 treatment after MCAO model. The washed Protein A/G agarose beads (P2055, Bioworld Biotechnology) was mixed with protein gently to wipe out the non‐specific binding of protein, and incubated with NEMO antibody (1 μg, ab178872, abcam) or IgG antibody (1 μg, 2729, Cell Signaling Technology) overnight at 4°C. Protein A/G agarose beads were added and incubated for 2 h on rotating wheel. The precipitate was washed with lysis buffer for three times and prepared for western blot.

### 2.12 Proximity ligation assay

The in situ proximity ligation assay (PLA) was performed according to the instruction (DUO92101, Sigma‐Aldrich). Primary microglia were blocked with Duolink® Blocking Solution for 60 min at 37°C, and then incubated with anti‐Ubiquitin (ab134953, abcam) and anti‐IKKγ (sc‐8032, Santa Cruz) overnight at 37°C. PLUS and MINUS PLA probes were diluted 1:5 in Duolink® Antibody Diluent and incubated with the microglia for 1 h. After washing with Wash Buffer A and applying the ligation solution for 30 min at 37°C, microglia were incubated with Amplification buffer for 100 min in dark. Images were taken with Olympus BX51 (Japan) fluorescence microscope and analyzed with ImageJ (ImageJ 1.5, NIH).

### 2.13 Transcriptome sequencing

Primary microglia were pretreated with 10 μM AZD1390 for 2 h and stimulated with LPS (100 ng/mL) for 24 h. Then total RNA was extracted with TRIzol (AG21101, Accurate Biology) and analyzed on a llluminaHiseq 2000 platform (Shanghai Majorbio Bio‐pharm Technology Co., Ltd). Differentially expressed genes (DEGs) were singled out with a adjust *p* ≤ 0.05 and change fold ≥2.0, subsequently performed the Gene Set Enrichment Analysis through Kyoto Encyclopedia of Genes and Genomes (KEGG).

### 2.14 Statistical analyses

Experimental data were analyzed with GraphPad Prism 8.0 software (GraphPad Software, USA) and expressed as the mean ± standard deviation. The normality of the data distribution was tested with Shapiro–Wilk test. If the data is normal distribution, Student's *t*‐test was applied to analyze the statistical significance between two groups, while the Mann–Whitney test was performed to compare the non‐normally distributed variables. For multiple groups, the one‐way analysis of variance (ANOVA) followed by Bonferroni's post hoc test or the Kruskal–Wallis test followed by Dunn's multiple comparison test was applied, and the statistical significance of data with two factors was analyzed by two‐way ANOVA followed by Tukey's post‐hoc test. *p* < 0.05 was regarded as statistically significant value.

## 3 RESULTS

### 3.1 AZD1390 ameliorates LPS‐induced inflammation in primary microglia

**FIGURE 1:**
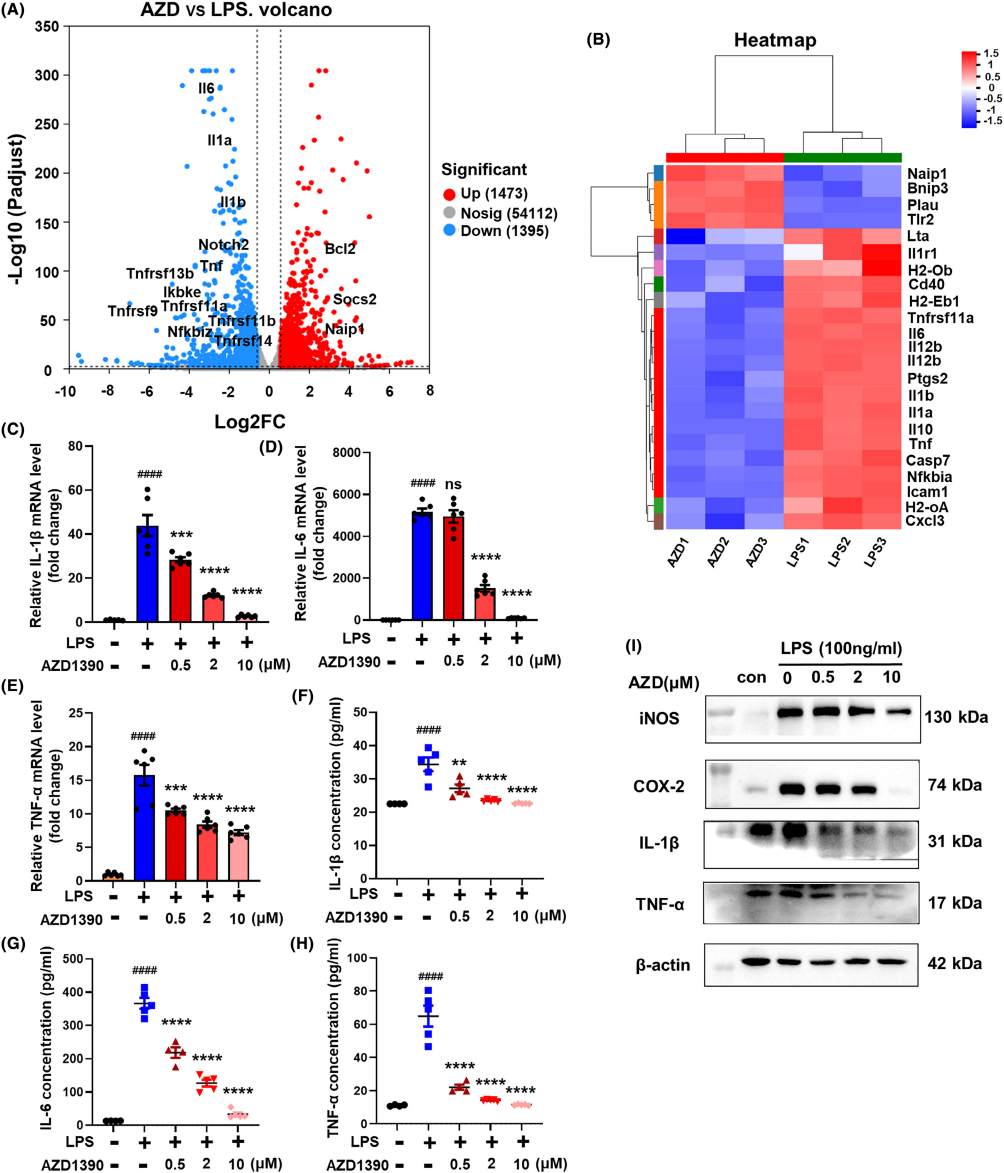
AZD1390 reduced the level of pro‐inflammatory cytokines in LPS‐stimulated primary microglia. (A, B) Primary microglia were pretreated with AZD1390 (10 mΜ) for 2 h and stimulated with LPS (100 ng/mL) for 24 h. Volcano plot of primary microglia treated with AZD1390 + LPS versus DMSO + LPS group. The numbers of differentially expressed genes (DEGs) were exhibited. *n* = 3 per group (A). Heat map showing gene expression changes of AZD1390 + LPS versus DMSO + LPS group in NF‐κB signaling pathway. *n* = 3 per group (B). (C–I) Primary microglia were pretreated with AZD1390 (0.5, 2, 10 mΜ) for 2 h and stimulated with LPS (100 ng/mL) for 24 h. The level of IL‐1β, IL‐6 and TNF‐α was detected by real‐time PCR after LPS treatment with or without AZD1390. *n* = 3 (C–E). The concentration of IL‐1β, IL‐6 and TNF‐α in the supernatants of primary microglia were measured via ELISA. *n* = 4–5 (F–H). The protein levels of iNOS, COX‐2, IL‐1β, IL‐6 and TNF‐α were analyzed with western blot and β‐actin was used as the internal reference. The values were presented as the means ± SEM. *n* = 3 per group (I). The data are presented as the mean ± SEM. *p*‐values were determined by the Kruskal–Wallis test with Dunn's post‐hoc analysis in (D)–(G). One‐way ANOVA with Tukey's post‐hoc analysis in (C) and (H). ^#^ *p* < 0.05, ^##^ *p* < 0.01, ^###^ *p* < 0.001, and ^####^ *p <* 0.0001 versus WT group; *p < 0.05, **p < 0.01, ***p < 0.001, and ****p < 0.0001 versus LPS‐treated group.

To determine the effect of AZD1390 in ischemic stroke, RNA‐sequencing analysis was performed and the results showed that multiple inflammatory cytokines such as IL‐1β, IL‐6, TNF‐α were downregulated in LPS‐induced microglia with AZD1390 pretreatment (Figure 1A,B). KEGG analysis showed that several pathways including TNF, PI3K‐Akt, NF‐κB and JAK–STAT signaling pathway were involved (Figure S1a), which indicated that AZD1390 might inhibit LPS induced neuroinflammation. To verify these data, the levels of IL‐1β, IL‐6 and TNF‐α were measured in AZD1390 treated microglia stimulated by LPS. The results showed that LPS upregulated the mRNA expression of IL‐1β, IL‐6 and TNF‐α, while pretreated with AZD1390 (0.5, 2 or 10 μM) could reverse these effects (Figure 1C–E). Moreover, the levels of IL‐1β, IL‐6 and TNF‐α in supernatant (Figure 1F–H) and protein levels of IL‐1β, TNF‐α, COX‐2 and iNOS (Figure 1I, Figure S1b–e) were greatly decreased in LPS‐induced microglia pretreated with AZD1390. These data suggested that AZD1390 ameliorated LPS‐induced inflammatory cytokines release in primary microglia.

### 3.2 AZD1390 attenuates ischemic brain injury and inflammation in MCAO mice

**FIGURE 2:**
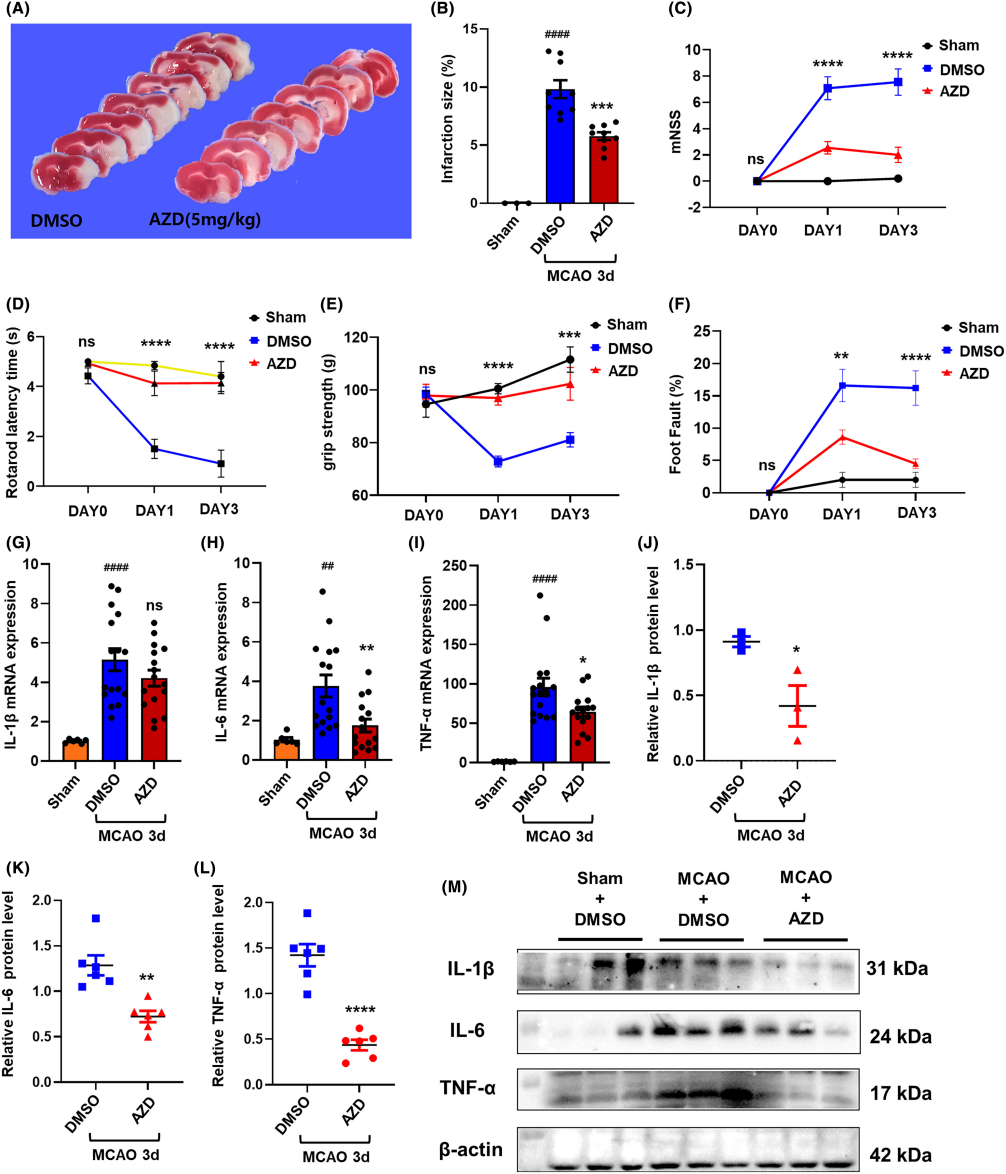
AZD1390 attenuates ischemic brain injury and inflammation in MCAO mice. (A, B) Mice were intraperitoneally injected with AZD1390 (5 mg/kg) after MCAO. TTC staining was represented at 3 days after MCAO. *n* = 9. (C–F) Mice were treated with AZD1390 (5 mg/kg) after MCAO and the mNSS test (C), rotarod test (D), grip test (E) and footfault test (F) were performed. *n* = 16–18 per group at MCAO 1 day, *n* = 15–16 per group at MCAO 3 days. (G–M) The levels of IL‐1β, IL‐6 and TNF‐α were measured with real‐time PCR (G–I, *n* = 6) and western blot (J–M, *n* = 3–6) at 3 days after MCAO and AZD1390 (5 mg/kg) treatment. The data are presented as the mean ± SEM. *p*‐values were determined by the Kruskal–Wallis test with Dunn's post‐hoc analysis in (H) and (I). One‐way ANOVA with Tukey's post‐hoc analysis in (B), (G), (J)–(L). Two‐way ANOVA with Tukey's post‐hoc analysis in (C)–(F). ^##^ *p* < 0.01, ^###^ *p* < 0.001 and ^####^ *p<* 0.0001 versus sham group; *p < 0.05, **p < 0.01, ***p < 0.001, and ****p < 0.0001 versus DMSO‐treated group.

**FIGURE 3:**
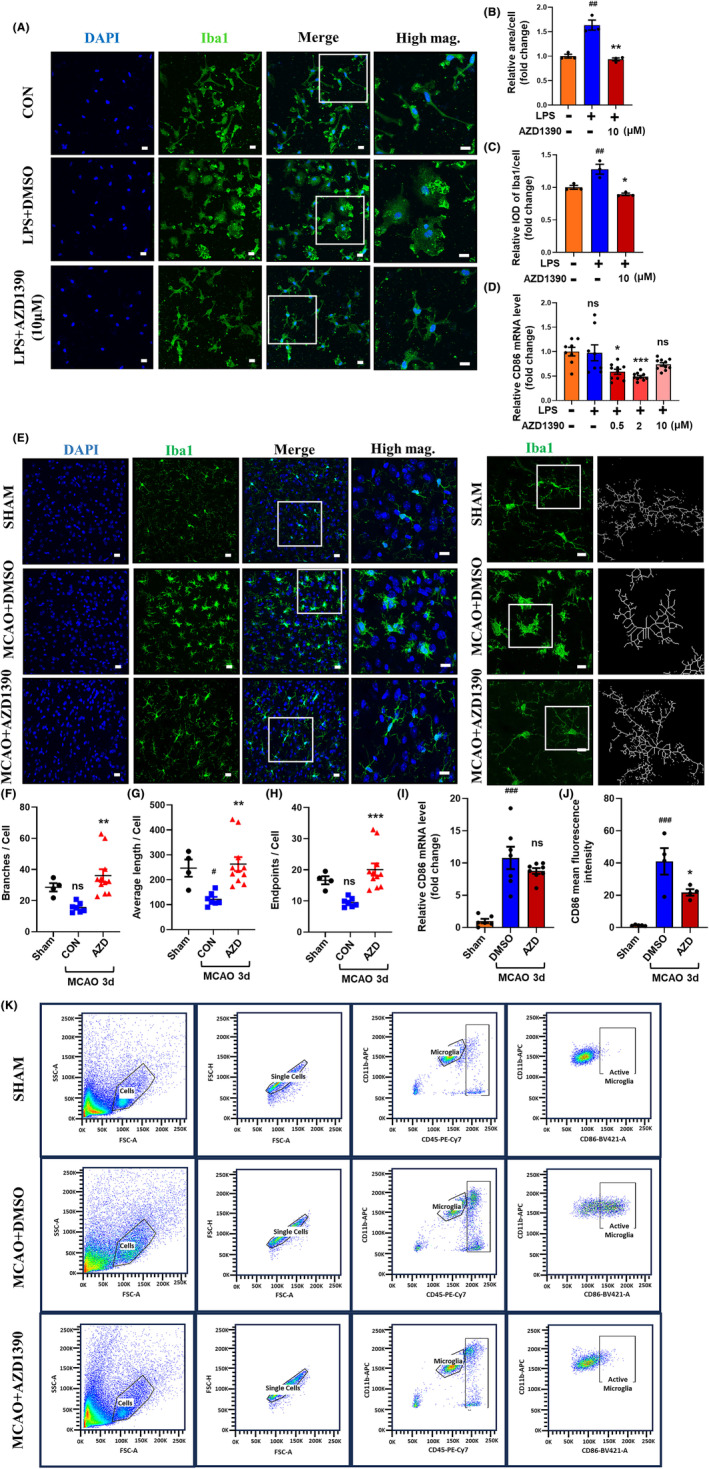
AZD1390 inhibited the activation of microglia in vivo and in vitro. (A–D) The primary microglia were pretreated with AZD1390 (10 mΜ) for 2 h and then stimulated with LPS for 24 h. The morphological feature was analyzed with immunocytochemistry using Iba1 antibody. The left 3 panels: Scale bar = 20 μm. The right panel: Scale bar = 10 μm (A). Surface area of cells and integrated optical density (IOD) of Iba1 in (A) was analyzed with ImageJ (B, C). The mRNA level of CD86 in primary microglia was measured with real‐time PCR. *n* = 5 (D). (E–H) Mice were treated with AZD1390 (5 mg/kg) for 3 days after MCAO, then immunofluorescence staining of the ischemic brain slices was performed, stained with DAPI (blue) and Iba1 (green). The left 3 panels: Scale bar = 20 μm. The right panel: Scale bar = 10 μm. *n* = 3 (E). The branches of microglia, endpoints of microglia, average length of branches, maximum length of branches were measured (*n* = 10, scale bar = 10 μm) (F–H). (I) The mRNA levels of CD86 was measured using real‐time PCR. *n* = 4. (J–K) The mean fluorescence intensity of CD86 was measured with flow cytometry at 3 days after MCAO and AZD1390 (5 mg/kg) treatment. *n* = 5. The data are presented as the mean ± SEM. *p*‐values were determined by the Kruskal–Wallis test with Dunn's post‐hoc analysis in (B), (F)–(H). One‐way ANOVA with Tukey's post‐hoc analysis in (C), (I), (J) and (D). ^#^ *p* < 0.05, ^##^ *p* < 0.01, ^###^ *p* < 0.001 and ^####^ *p <* 0.0001 versus WT group; *p < 0.05, **p < 0.01, ***p < 0.001, and ****p < 0.0001 versus LPS/DMSO‐treated group.

To explore the therapeutic effect of AZD1390 in ischemic stroke, AZD1390 was intraperitoneally injected after MCAO modeling, and behavioral tests and TTC staining were conducted. The data showed that AZD1390 administration remarkably reduced the infarct volume in MCAO mice (Figure 2A,B, Figure S1f,g). In addition, AZD1390 treatment could improve mNSS, rotarod latency time, grip strength and foot fault tests (Figure 3C–F, Figure S1h–u). Consistently, MCAO upregulated the mRNA and protein levels of IL‐1β, IL‐6 and TNF‐α in penumbra, and AZD1390 could reverse these effects (Figure 2G–M). It was noteworthy that the 5 mg/kg group showed the best therapeutic effect. All these results indicated that AZD1390 alleviated the ischemic brain injury and neurological deficits in ischemic stroke mice.

### 3.3 AZD1390 inhibits the activation of microglia in vitro and in vivo

Since AZD1390 reduced the expression of pro‐inflammatory cytokines, we next explored the effects of AZD1390 on the morphological of microglia. LPS stimulated microglia displayed swollen soma and more enhanced Iba1 fluorescence, which was partially rescued by AZD1390 pretreatment (Figure 3A–C). Moreover, the mRNA expression of CD86 was increased after LPS treatment, while AZD1390 could attenuate these effects (Figure 3D). To determine whether AZD1390 inhibit microglia activation after MCAO modeling, immunofluorescent staining was performed and the results indicated that AZD1390 shifted microglia from active state to the relatively quiescent state, as represented by more branches and endpoints, longer maximum and average length (Figure 3E–H). Moreover, the mRNA level of CD86 and the proportion of CD86+ microglia were significantly increased after MCAO, while AZD1390 could reverse these effects (Figure 3I–K). Therefore, these results showed that AZD1390 inhibited microglia activation in vitro and in vivo.

### 3.4 AZD1390 inhibits the NF‐κB signaling pathway in LPS‐induced microglia and ischemic stroke

**FIGURE 4:**
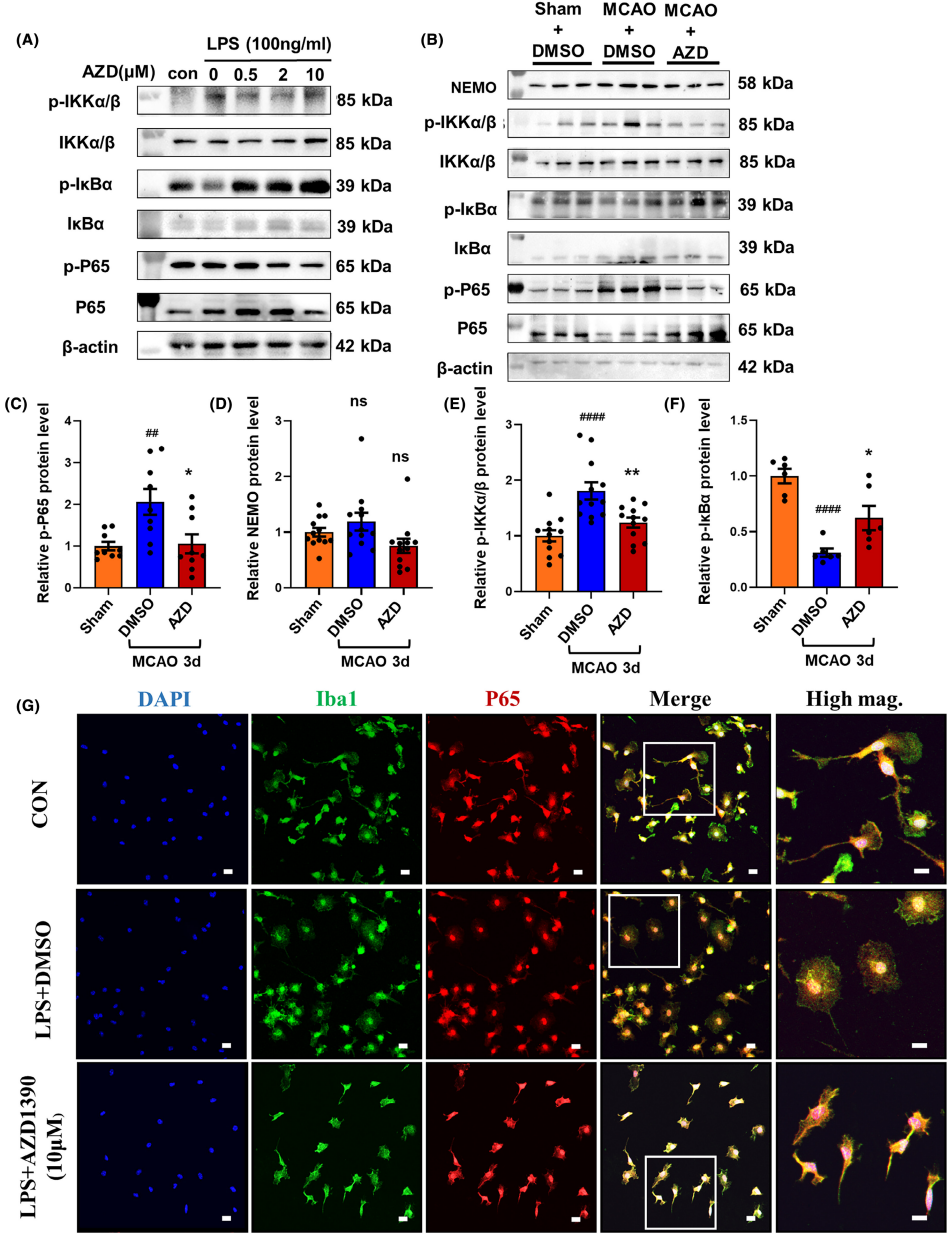
AZD1390 inhibits NF‐κB signaling pathway in LPS‐induced microglia and ischemic stroke. Mice were intraperitoneally injected with AZD1390 (5 mg/kg) after MCAO. The primary microglia were pretreated with AZD1390 for 2 h and then stimulated with LPS for 24 h. (A–E) The relative protein levels of NEMO, p‐IKKα and β/IKKα and β, p‐IκBα/IκBα, p‐NF‐κBp65/NF‐κBp65 were detected with western blot in vivo (*n* = 3, A) and in vitro (*n* = 12, B–F). (G) The primary microglia treated with AZD1390 (10 mΜ) + LPS or DMSO + LPS were stained with DAPI (blue), Iba1 (green) and p65 (red). The left 4 panels: Scale bar = 20 μm. The right panel: Scale bar = 10 μm. These values are expressed as mean ± SEM. *p*‐values were determined by the Kruskal–Wallis test with Dunn's post‐hoc analysis in (C), (D) and (F). One‐way ANOVA with Tukey's post‐hoc analysis in (E). ^##^ *p* < 0.01, ^###^ *p* < 0.001 and ^####^ *p <* 0.0001 versus WT group; *p < 0.05, **p < 0.01, ***p < 0.001, and ****p < 0.0001 versus LPS/DMSO‐treated group.

NF‐κB signaling was identified as a most dysregulated pathway in AZD1390 treated microglia (Figure S1a). To clarify the critical role of NF‐κB signaling pathway in AZD1390 mediated antineuroinflammatory effects, the activation of NF‐κB signaling pathway was determined by western blot and immunofluorescent staining. The ratio of phosphorylated p65/p65, phosphorylated IKKα and β/IKKα and β was reduced, while the ratio of phosphorylated IκBα/IκBα was increased in AZD1390 pretreatment microglia and in the penumbra of MCAO mice (Figure 4A–F, Figure S2a–i). Moreover, LPS induced the nuclear translocation of NF‐κB, and pretreated with AZD1390 could reverse these effects in primary microglia (Figure 4G). These results indicated that AZD1390 inhibited the NF‐κB signaling pathway in LPS‐induced microglia and MCAO mice, which might contribute to the anti‐inflammatory effect of AZD1390.

### 3.5 AZD1390 decreases the ATM‐dependent ubiquitylation of NEMO to suppress the NF‐κB signaling pathway

**FIGURE 5:**
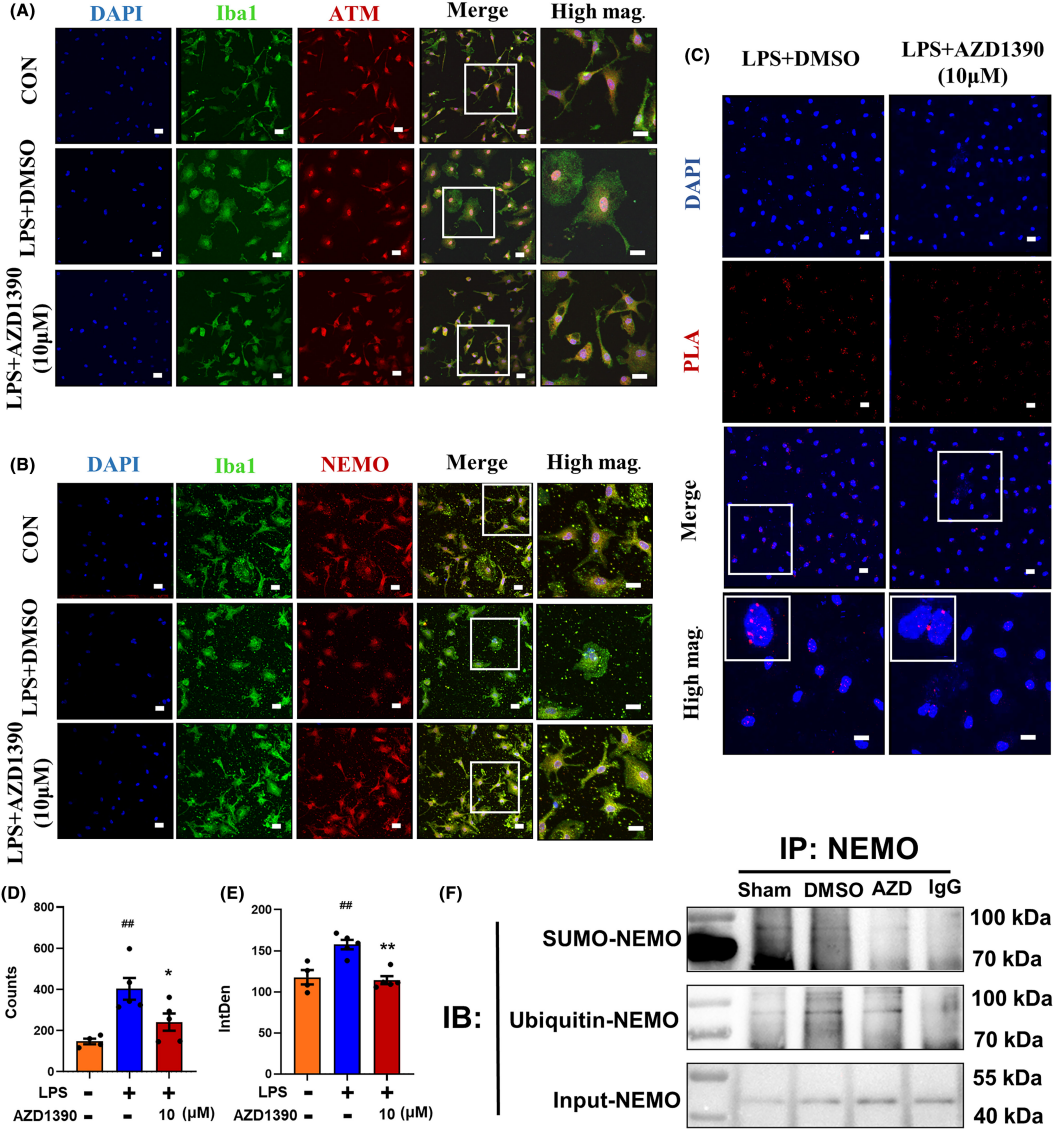
AZD1390 decreased the ATM‐dependent ubiquitylation of NEMO to suppress NF‐κB signaling pathway. The primary microglia were pretreated with AZD1390 (10 mΜ) for 2 h and then stimulated with LPS for 24 h. (A, B) The primary microglia were stained with DAPI (blue), Iba1 (green), ATM (red, upper) and NEMO (red, lower) after being treated with AZD1390 + LPS or DMSO + LPS. The left 4 panels: Scale bar = 20 μm. The right panel: Scale bar = 10 μm. (C–E) The proximity ligation assay was performed in primary microglia treated with AZD1390 + LPS or DMSO + LPS, and the counts and fluorescence intensity of PLA signals were detected using ImageJ. (F) Protein was extracted from the brain penumbra tissue with or without AZD1390 (5 mg/kg) treatment, and immunoprecipitated with NEMO antibody and analyzed using western blot with anti‐SUMO‐1 or anti‐ubiquitin antibody. These values are expressed as mean ± SEM. *p*‐values were determined by the Kruskal–Wallis test with Dunn's post‐hoc analysis in (E). One‐way ANOVA with Tukey's post‐hoc analysis in (D). ^##^ *p* < 0.01, ^###^ *p* < 0.001 and ^####^ *p <* 0.0001 versus sham group; *p < 0.05, **p < 0.01, ***p < 0.001, and ****p < 0.0001 versus LPS‐treated group.

Given that ATM/NEMO played an important role in regulating NF‐κB signaling pathways, immunofluorescent staining was performed and the results showed that AZD1390 restricted ATM nuclear translocation in LPS‐treated microglia (Figure 5A). Notably, AZD1390 suppressed NEMO cytoplasm translocation (Figure 5B), indicating that AZD1390 might reduce the ATM‐mediated translocation of NEMO and decrease the activation of subsequent downstream factors. In addition, the results of PLA showed that the ubiquitylation of NEMO was elevated after LPS stimulation and AZD1390 decreased NEMO ubiquitylation (Figure 5C–E). Furthermore, the immunoprecipitation data showed that both the levels of Ub‐NEMO and SUMO‐NEMO were reduced by AZD1390 pretreatment (Figure 5F). These results showed that AZD1390 decreased the ATM‐independent sumoylation and ATM‐dependent ubiquitylation of NEMO to suppress NF‐κB signaling pathway.

## 4 DISCUSSION

In current study, we found that: (1) AZD1390 reduced the expression of proinflammatory cytokines in vitro and in vivo, decreased the infarct volume and rescued the neurological deficits in MCAO mice; (2) AZD1390 alleviated the activation of microglia and suppressed NF‐κB signaling pathway; (3) AZD1390 reduced the nuclear translation of ATM and depressed the SUMO attachment and ubiquitylation of NEMO, which restricted the nuclear exportation of NEMO to inhibit the NF‐κB pathway. Therefore, to the best of our knowledge, for the first time, we have demonstrated that AZD1390 attenuated microglia‐mediated neuroinflammation in ischemic stroke, which might be associated with the suppression of NF‐κB signaling pathway.

While stroke occurs, microglia is activated and recruited rapidly to defend against neurologic impairment.^8^ However, inappropriate activation of microglia leads to the release of neurotoxic factors such as IL‐1β, IL‐6, TNF‐α, iNOS and COX‐2, which sparks series events of neuronal cell injury.^9^, ^10^ Accumulative studies have verified the vital role of microglia in post‐stroke inflammation. Classically activated (M1) microglia is assumed to release pro‐inflammatory cytokines and induce brain damage, while alternatively activated (M2) microglia is thought to release anti‐inflammatory cytokines and provide neuroprotective effect.^11^, ^12^ Argon promotes the switch of M1 towards M2 microglia/macrophage polarization and reduces brain inflammation after stroke.^13^ In addition, elevated homocysteine level aggravates the brain damage through exaggerating microglia activation and inflammation.^14^, ^15^ Moreover, our group have shown that acute ischemia spatially and transcriptionally induces microglial subclusters. Ischemic core‐associated microglia induce excessive neuroinflammation and ischemic penumbra‐associated microglia probably exhibit neuroprotective features.^16^ Here, in this study, we have showed that AZD1390 inhibited the microglia activation and reduced the level of pro‐inflammatory cytokines, which rescued the brain damage after ischemic stroke.

NF‐κB is thought to be a master regulatory factor in microglia‐mediated inflammatory response.^17^ Under the normal state, NF‐κB normally combines with IκB protein complex to keep inactive in the cytoplasm. However, the activation of IKKα/β induces IκBα phosphorylation and degradation, which allows the liberation of NF‐κB p65 subunit and translocation to nuclear to release the pro‐inflammatory cytokines in microglia.^18^ A growing body of research has suggested NF‐κB signaling as a potential therapeutic target in attenuating inflammation and brain damage in ischemic stroke. Aloe‐emodin prevents nerve injury and neuroinflammation via NF‐κB pathway in ischemic stroke.^19^ Ischemia reperfusion induces astrocytic inflammation and neuronal oxidative injury, while pterostilbene treatment rescues these effects by inhibiting NF‐κB phosphorylation.^20^ TDP‐43 has been identified as a disease‐associated protein and might play a potentially pathogenic role as co‐activator of NF‐κB in brain injuries.^21^, ^22^ The age‐related deregulation of TDP‐43 enhances NF‐κB activation and exaggerates the inflammation and neuronal damage.^23^ A novel inhibitor of NF‐κB, rographolide, suppresses NF‐κB‐mediated inflammation and shows neuronal protective effects on ischemic stroke.^24^ Our previous data showed that imperatorin suppresses MAPK and NF‐κB pathway to alleviate neurological deficits and reduces the infarct volume.^5^ Here, we showed that AZD1390 suppressed the NF‐κB pathway and attenuated post‐ischemic inflammation and neurological deficits in MCAO mice.

NEMO, the regulatory subunit of IKK complex, plays a central role in NF‐κB signaling pathway.^7^, ^25^ NEMO is encoded by the X‐linked IKBKG/NEMO gene and the IKBKG/NEMO mutation leads to immunodeficiency and inflammation.^26^, ^27^ While sensing the stimulators, NEMO translocates to nuclear via site‐specific SUMO‐attachment. Subsequently, NEMO is ubiquitylated and exported to cytoplasm, which phosphorylates IKK complex and finally actives NF‐κB pathway. It should be noted that ATM is indispensable for ubiquitylation of NEMO.^28^ ATM is a vital signal‐transducing kinase for mediating certain forms of DNA damage, and deficiency of ATM delays the inflammatory response such as the accumulation of neutrophils and macrophages post myocardial infarction.^29^, ^30^ And NEMO ubiquitylation is completely compromised without ATM.^7^ Previous studies have confirmed the essential role of polyubiquitin chains (polyUb) binding by NEMO in IKK activation.^7^, ^31^ Furthermore, polyUb binds to NEMO to form the liquid‐like droplets under the stimulators, which subsequently activates the IKK complex, while impairment of NEMO liquid‐like condensates suppresses NF‐κB activation and induces immunodeficiency and inflammation.^32^ In this study, we found that AZD1390, a specific ATM inhibitor, could reduce the ubiquitylation of NEMO to restrict its nuclear exportation and suppress the activation of NF‐κB. Surprisingly, AZD1390 reduced the SUMO attachment of NEMO, and we speculated that there were at least 3 reasons: (1) AZD1390 attenuated the DNA damage to suppress the sumoylation of NEMO. Previous studies showed that DNA damage agents induced the DNA double strand break and NEMO sumoylation, which activated ATM and subsequently induced the ubiquitination of NEMO.^7^, ^25^ (2) AZD1390 induced the deconjugation of SUMO. It was previously implicated that YopJ, one of the Yersinia effectors, could decrease the cellular concentration of SUMO‐1‐conjugated proteins and the free SUMO‐1 in inhibition of cytokine‐induced NF‐κB activation.^33^ (3) AZD1390 might disrupt the sumoylation sites on NEMO. Two lysine residues of NEMO, K277 and K309, were necessary for sumoylation and ubiquitylation of NEMO.^7^ Disruption of the sites leads to the reduction of SUMO‐ and Ub‐NEMO and suppressed the NF‐κB activation. Further mechanistic studies will be needed in the following studies.

In summary, our study demonstrated that AZD1390 alleviated ischemic brain injury in experimental stroke and attenuated the activation of microglia and neuroinflammation, which might attribute to the suppression of the NF‐κB signaling pathway through NEMO modification. Thus, these results indicated that AZD1390 might be an attractive agent for the treatment of ischemic stroke.

## AUTHOR CONTRIBUTIONS

Concept: XLZ and YX. Research design: ZL and XLZ. MCAO modeling: SNX and HYY Primary cell culture: XYB. Data collection: ZL, LJQ, YL, LQC, SX, ZWX and JH. Data analysis and interpretation: ZL, LJQ, YL, LQC, JWG and ZWX. Manuscript writing: ZL. Manuscript revision: XLZ and YX. All authors approved the final version of the manuscript.

## CONFLICT OF INTEREST STATEMENT

Xu, Yun is an Editorial Board member of the CNS Neuroscience and Therapeutics and a co‐author of this article. To minimize the bias, they were excluded from all editorial decision‐making related to the acceptance of this article for publication. The other authors declare that there are no conflicts of interest associated with this article.

## Supporting information


Figures S1–S2

## Data Availability

The data that support the findings of this study are available from the corresponding author upon reasonable request.
