# Supplementary material for: AZD1390, an ataxia telangiectasia mutated inhibitor, attenuates microglia‐mediated neuroinflammation and ischemic brain injury
Source: CNS Neurosci Ther. 2024 Apr 26;30(4):e14696. doi: 10.1111/cns.14696 (PMC11048048; doi:10.1111/cns.14696)
Supplement: Supplementary file 1 — Figures S1–S2 [file CNS-30-e14696-s001.zip › Legends.docx]

**Figure S1** (a) Primary microglia were pretreated with AZD1390 (10 mΜ) for 2 h and stimulated with LPS (100 ng/ml) for 24 h. The KEGG enrichment analysis of primary microglia treated with AZD1390 + LPS versus DMSO + LPS group were exhibited. n = 3 per group. (b-e) Primary microglia were pretreated with AZD1390 (0.5, 2, 10 mΜ) for 2 h and stimulated with LPS (100 ng/ml) for 24 h. The level of IL-1β, IL-6 and TNF-α was detected by Western blot after LPS treatment with or without AZD1390. n = 3. Mice were intraperitoneally injected with different concentrations of AZD1390 after MCAO. TTC staining was represented at 3 days after MCAO. n = 5 (f-g). The results of rotarod test (h-j) and grip strength test (k-m) were measured in MCAO mice after AZD1390 (2/5/10mg/kg) treatment. n = 5-6 per group at MCAO 1 d, n = 5-6 per group at MCAO 3 d. The results of mNSS (n-o), rotarod test (p-q), grip strength test (r-s) and foot fault (t-u) were measured in MCAO mice after AZD1390 (5mg/kg) treatment. n = 16-18 per group at MCAO 1 d, n = 15-16 per group at MCAO 3 d. These values are expressed as mean ± SEM. p-values were determined by the Kruskal–Wallis test with Dunn’s post-hoc analysis in (d), (e), (h), (i), (n), (o), (p) and (q). One-way ANOVA with Tukey’s post-hoc analysis in (b), (c), (g), (k), (l), (r), (s), (t) and (u). Two-way ANOVA with Tukey’s post-hoc analysis in (j) and (m). ##p < 0.01, ###p < 0.001vs. control group; *p < 0.05, **p < 0.01, ***p < 0.001, ****p < 0.0001 vs. LPS-treated group.

**Figure S2** (a-f) Primary microglia were pretreated with AZD1390 (0.5, 2, 10 mΜ) for 2 h and stimulated with LPS (100 ng/ml) for 24 h. The relative protein levels of NF-κBp65, IKKα and β, IκBα, p-NF-κBp65/NF-κBp65, p-IKKα and β/IKKα and β, p-IκBα/IκBα were detected with western blot. n = 3. (g-i) Mice were intraperitoneally injected with AZD1390 (5mg/kg) after MCAO. The relative protein levels of NF-κBp65, IKKα and β, IκBα were detected with western blot. n = 3. These values are expressed as mean ± SEM. p-values were determined by the Kruskal–Wallis test with Dunn’s post-hoc analysis in (f), (h) and (i). One-way ANOVA with Tukey’s post-hoc analysis in (a), (b), (c), (d), (e) and (g). ##p < 0.01, ###p < 0.001vs. WT group; *p < 0.05, **p < 0.01, ***p < 0.001, ****p < 0.0001 vs. LPS/DMSO-treated group.
