# Supplementary figures and images for: AZD1390, an ataxia telangiectasia mutated inhibitor, attenuates microglia‐mediated neuroinflammation and ischemic brain injury
Source: CNS Neurosci Ther. 2024 Apr 26;30(4):e14696. doi: 10.1111/cns.14696 (PMC11048048; doi:10.1111/cns.14696)

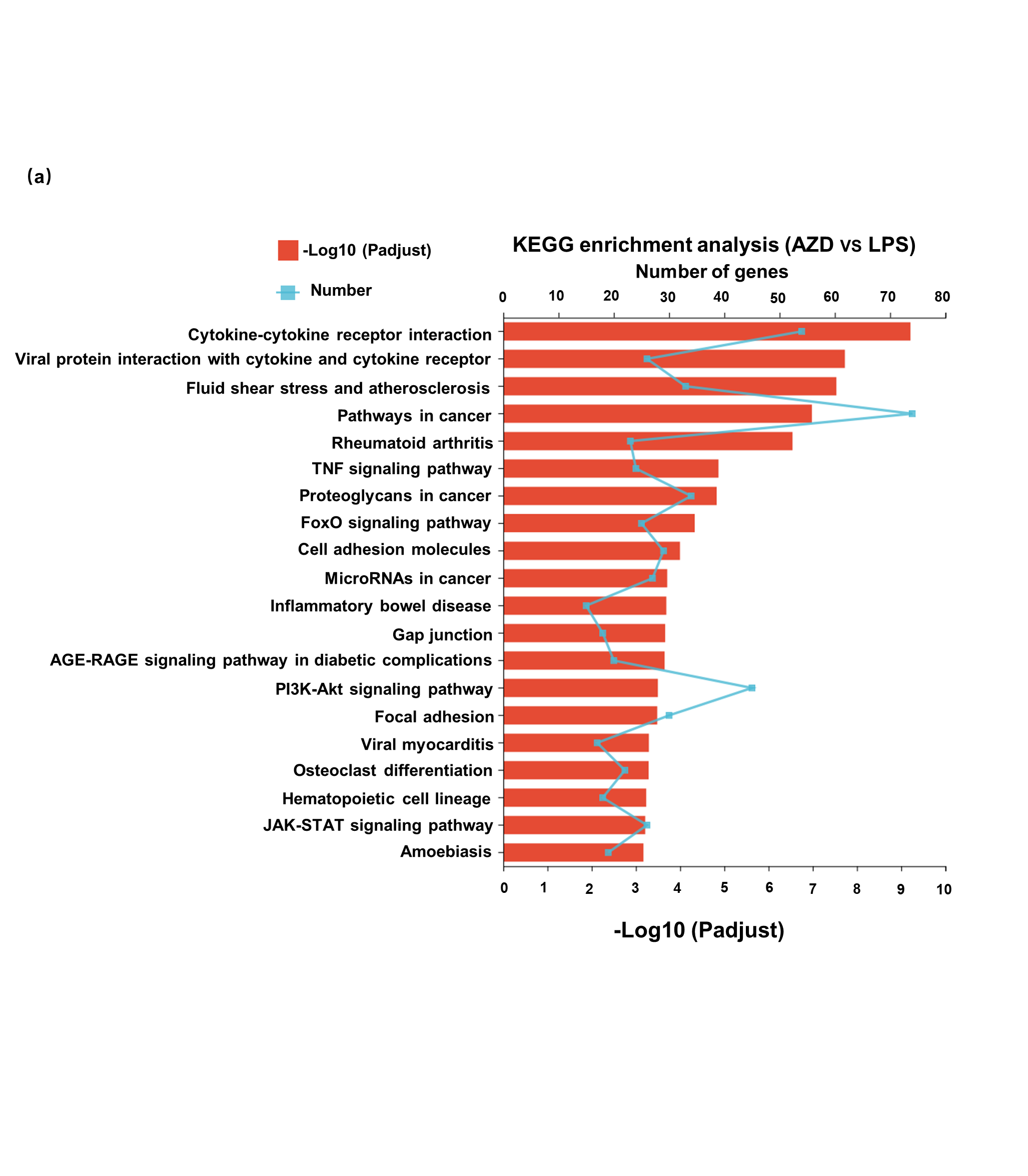

Supplement: Supplementary file 1 — Figures S1–S2 [file CNS-30-e14696-s001.zip › FigureS1.1.TIF]

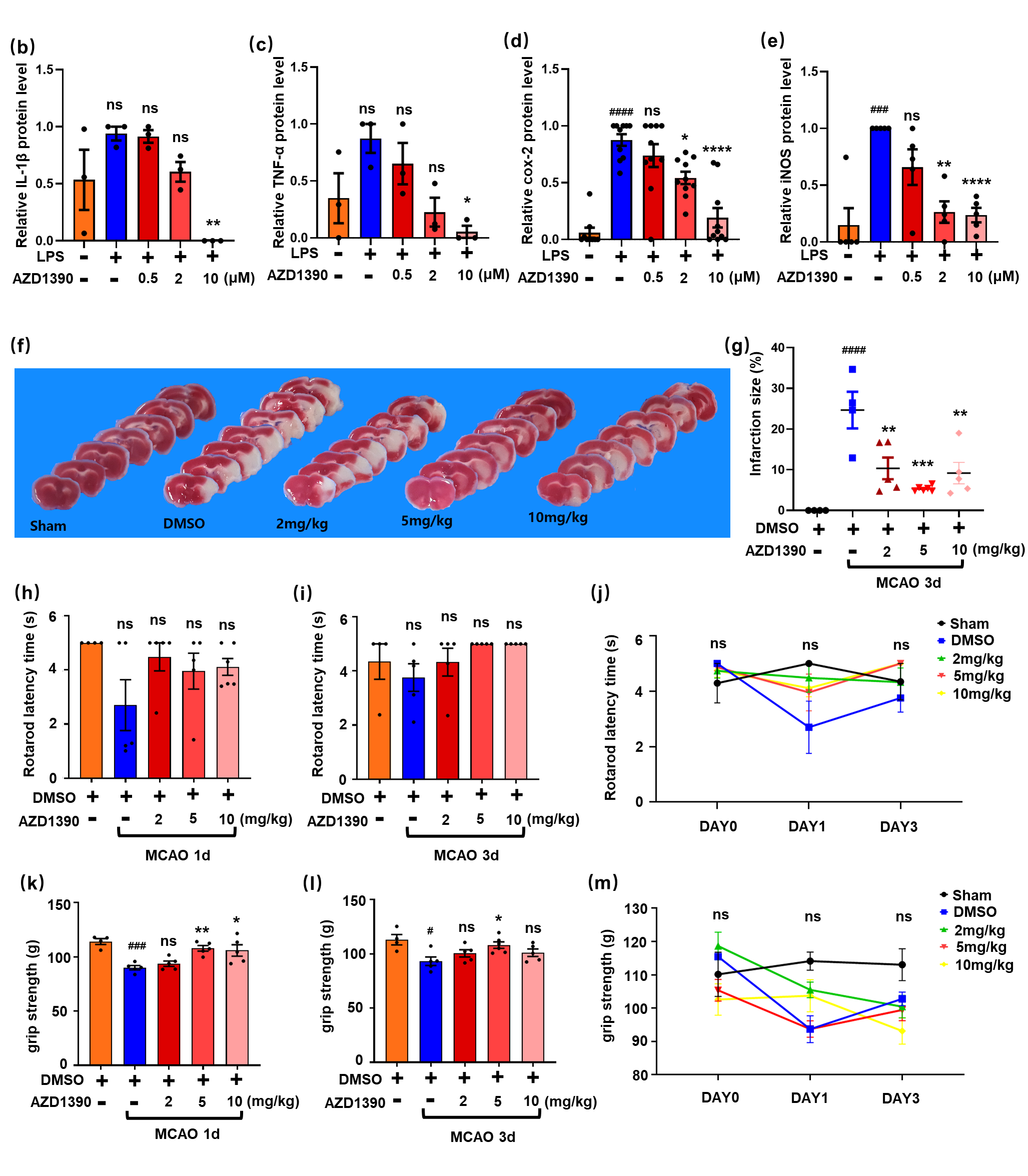

Supplement: Supplementary file 1 — Figures S1–S2 [file CNS-30-e14696-s001.zip › FigureS1.2.TIF]

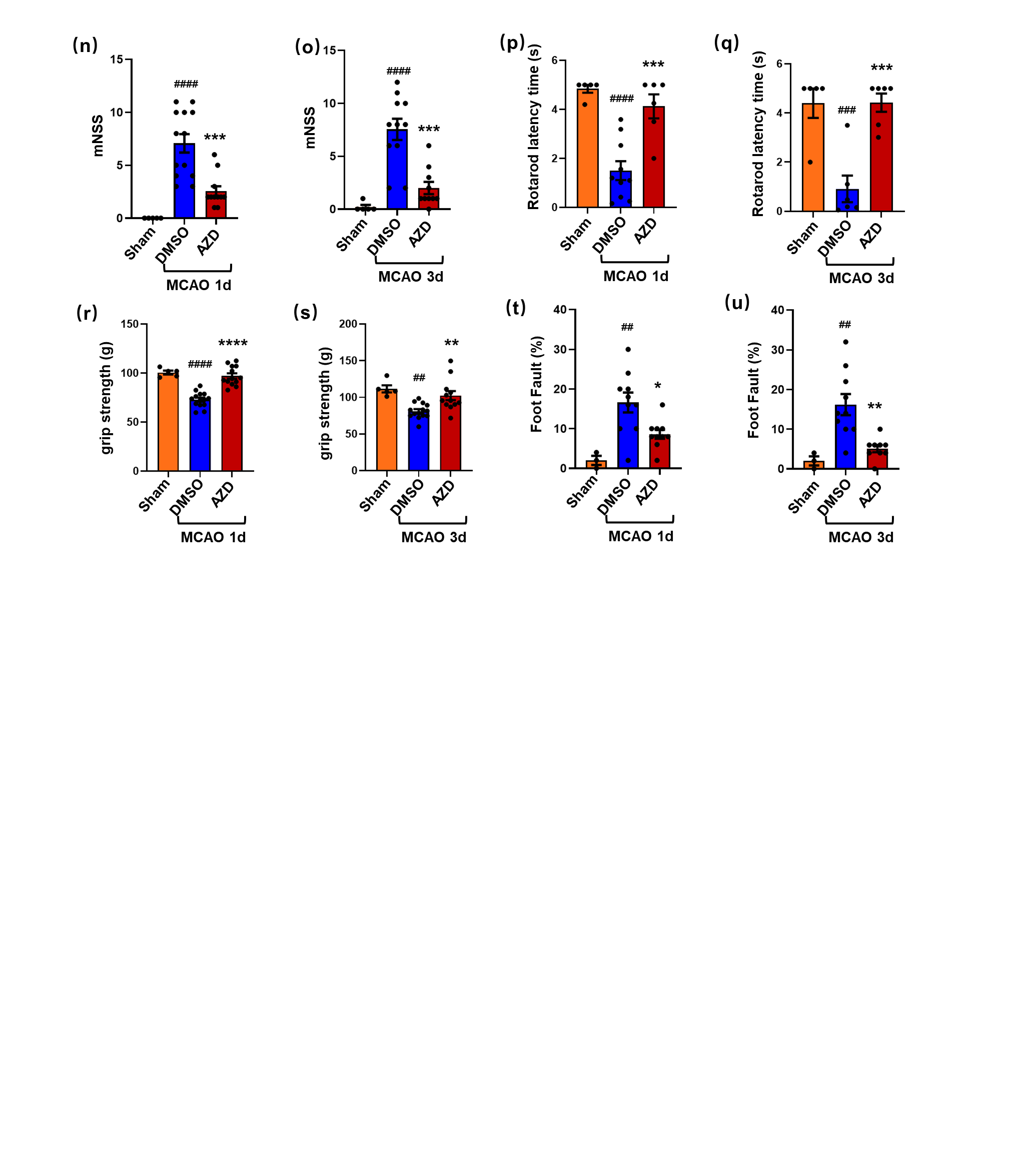

Supplement: Supplementary file 1 — Figures S1–S2 [file CNS-30-e14696-s001.zip › FigureS1.3.TIF]

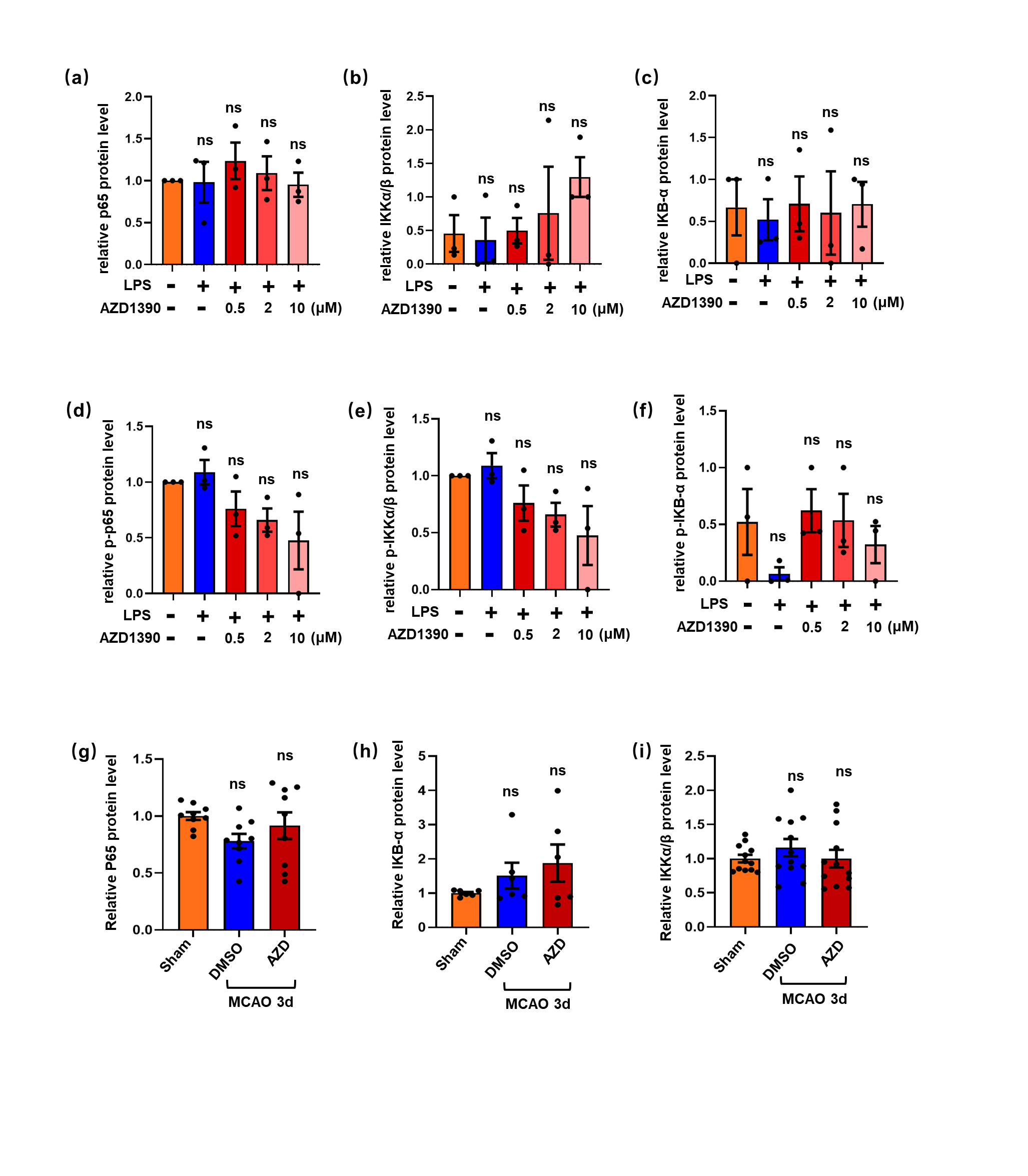

Supplement: Supplementary file 1 — Figures S1–S2 [file CNS-30-e14696-s001.zip › FigureS2.TIF]
